# Genetic adaptation of the antibacterial human innate immunity network

**DOI:** 10.1186/1471-2148-11-202

**Published:** 2011-07-11

**Authors:** Ferran Casals, Martin Sikora, Hafid Laayouni, Ludovica Montanucci, Aura Muntasell, Ross Lazarus, Francesc Calafell, Philip Awadalla, Mihai G Netea, Jaume Bertranpetit

**Affiliations:** 1 Institute of Evolutionary Biology (UPF-CSIC), CEXS - UPF - PRBB, Barcelona, Catalonia, Spain; 2 Centre de Recherche, CHU Sainte-Justine, Université de Montréal, Montréal, Québec H3T 1C5, Canada; 3 Unitat d'Immunologia, IMIM-Hospital del Mar, Barcelona, Catalonia, Spain; 4 Channing Laboratory, Department of Medicine, Brigham and Women's Hospital, Harvard Medical School, Boston, MA 02115, USA; 5 Department of Medicine, Radboud University Nijmegen Medical Center, 6500 HB Nijmegen, The Netherlands; 6 Department of Genetics, Stanford University School of Medicine, Stanford, USA

## Abstract

**Background:**

Pathogens have represented an important selective force during the adaptation of modern human populations to changing social and other environmental conditions. The evolution of the immune system has therefore been influenced by these pressures. Genomic scans have revealed that immune system is one of the functions enriched with genes under adaptive selection.

**Results:**

Here, we describe how the innate immune system has responded to these challenges, through the analysis of resequencing data for 132 innate immunity genes in two human populations. Results are interpreted in the context of the functional and interaction networks defined by these genes. Nucleotide diversity is lower in the adaptors and modulators functional classes, and is negatively correlated with the centrality of the proteins within the interaction network. We also produced a list of candidate genes under positive or balancing selection in each population detected by neutrality tests and showed that some functional classes are preferential targets for selection.

**Conclusions:**

We found evidence that the role of each gene in the network conditions the capacity to evolve or their evolvability: genes at the core of the network are more constrained, while adaptation mostly occurred at particular positions at the network edges. Interestingly, the functional classes containing most of the genes with signatures of balancing selection are involved in autoinflammatory and autoimmune diseases, suggesting a counterbalance between the beneficial and deleterious effects of the immune response.

## Background

Infectious diseases are among the most important selective agents for any vertebrate species. In humans, they have represented a great challenge in our adaptation to new environments and social practices, with increasing population densities sustained by improved agricultural technologies and cattle domestication favoring their emergence and spread in the last ten thousand years. The human immune system seems likely to have played a key role in the adaption of the different populations to changing conditions and emerging infections. This hypothesis is supported by recent genomic scans for signatures of adaptive selection in human populations showing that immune function is one of the classes enriched with genes under positive or balancing selection [1-6], the two evolutionary forces underlying adaptation. The literature includes a large collection of human genes related to the host-pathogen interaction with signatures of adaptation [7,8]. Vertebrate immune function can be divided into the adaptive, exclusive of this phylogenetic group, and the ancient innate immune system, common to most multicellular organisms. Innate immunity constitutes the first barrier of defense, and acts in a semi-specific way by recognizing pathogen-associated molecular patterns (PAMPs), which are essential and conserved components of the pathogens. Substantial evidence of positive or balancing selection acting on some of these genes has been reported [9-15].

In this manuscript, we address the analysis of the footprint of the adaptive selection in the innate immune mechanisms involved in (mostly) antibacterial host defense. Bacteria have probably been the most important human pathogens, with a major impact on morbidity and mortality. Although some of the pathways described here are also involved in host defense against human parasites, fungi and viruses, the pattern recognition receptor (PRR) gene families analyzed (TLRs and NLRs) are the most important for non-adaptive recognition of bacteria. The other two major classes of PRR gene families involved in the host defense to fungi (C-type lectins) and viruses (RiG-I helicases) are not included in the present study.

There is an increasing interest in characterizing the evolutionary dynamics of proteins in the context of their functional network. Published data on biological networks, for example in the form of experimentally derived data sets of protein-protein interactions, or curated databases of functional pathways, have facilitated studies relating evolutionary parameters (mainly the footprint of natural selection) to network topology [16-19]. We use molecular signatures in particular genes to explore adaptation of the functional network, seeking whether human adaptation at the molecular level of the innate immunity system has occurred preferentially on some functional class or classes (with more plasticity to respond to the pathogenic pressures) or in specific positions of the network structure. The final goal of this work is to unravel past selective forces that have shaped human immunological responses to bacterial infections having acted in humans; the approach is based on detecting adaptations through natural selection uncovered in resequencing information in two populations (Europeans and Africans). The results are interpreted in the functional context of gene products interacting in well-identified networks.

## Methods

### Selection of genes and DNA sequences

**Figure 1:**
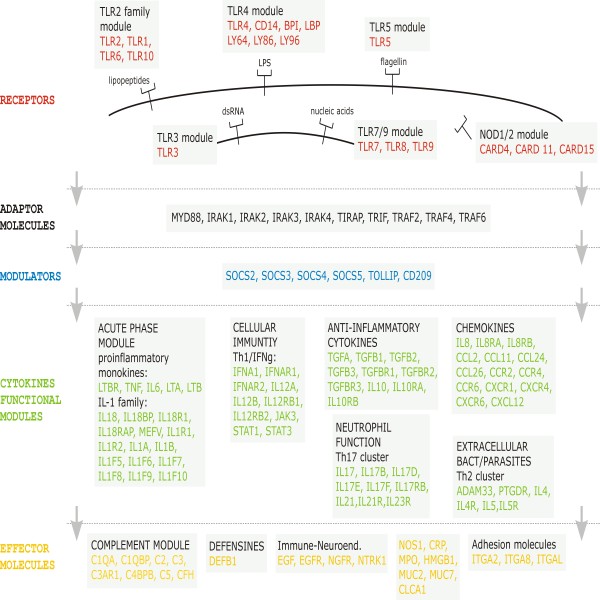
**Genes included in the study**. Schematic representation of the function on the innate immune system of the 132 genes analyzed in this work. The ligands of the receptors are also showed.

We included in the study as many genes as possible involved in innate immunity with available resequencing data. Data for 132 genes were retrieved from different sources. Sixty-two genes were from the Innate Immunity Program in Genomics Applications (IIPGA, http://innateimmunity.com[20]), only considering those genes with available information on the sequencing primers used. In addition, data for seven genes was obtained from Environmental Genome Project database (NIEHS SNPs, http://egp.gs.washington.edu), and for 54 from the SeattleSNPs database (http://pga.gs.washington.edu). All sequence data available, both from coding and noncoding regions, was included in the analysis. For the analysis, we also included the previously published results [11] for eight additional genes from Innate Immunity Program in Genomics Applications and one from the SeattleSNPs database. Genes were classified according to their function into a comprehensive functional network (Figure 1). Chimpanzee sequences (*panTro2*) were obtained from GenBank (http://www.ncbi.nlm.nih.gov/) and Ensembl database (http://www.ensembl.org/index.html) to be used as outgroups. For *IL1F7 *we used as outgroup the sequence from *Pongo pygmaeus *from the Ensemble database. Sequence alignments were performed with ClustalW [21]. Divergence estimations were retrieved from BioMart database (http://uswest.ensembl.org/biomart/index.html), only for unequivocal 1-to-1 hits to functional sequences. dN/dS values correspond to the comparison of humans and *Pan troglodytes *for all genes except TLR5 and IL1F8 (*Gorilla gorilla*) and *IL1F7 *(*Pongo pygmaeus*).

All included genes from the Innate Immunity Program in Genomics Applications, and 44 from SeattleSNPs were resequenced in the same 23 European-American and 24 African-American samples included in the Coriell CEPH/African American panel (Coriell Institute for Medical Research, Camden, NJ). The remaining 11 genes from Seattle SNPs were resequenced in 23 European (HapMap CEU) and 24 African individuals (HapMap YRI) individuals. For the seven genes from the Environmental Genome Project database we retrieved resequencing data in 22 European (HapMap CEU) and 15 Coriell African American individuals.

### Molecular Data Analysis

The following diversity statistics and neutrality tests were calculated for every gene: heterozygosity or π [22], Tajima's D [23], Fu and Li's F*, D*, F and D [24] and the normalized Fay and Wu's H tests [25] using DnaSP v5 [26] and MANVa [27]. Indels and triallelic positions were not included in the analyses. Genes with less than five segregating sites (*MYD88 *in Africans; *MYD88, IL5 *and *TLR3 *in Europeans) where not considered in the neutrality tests analysis. COSI software [28] was used to calculate the significance of neutrality tests by means of coalescent simulations, using the model which takes into consideration the demographic history of humans. COSI produces data that closely resembles empirical data from African American, West African and European populations. 1,000 replicates were performed by using the local recombination rate estimate of each region obtained from HapMap (http://www.hapmap.org). In the case of *C3 *and *ITGA8 *it was not possible to run the simulations because of its length and high recombination rate. The excess of genes in a given functional class under positive, balancing or both selection was tested by means of Fisher's Exact test in a two by two table including: the number of genes in this particular functional class under selection, the number of genes in this particular functional class without evidences of selection, the number of remaining genes (not in this functional class) under selection, and the number of remaining genes (not in this functional class) without evidences of selection.

### Network analysis

We used the MiMI plugin [29] for Cytoscape [30] to retrieve all known interactions for the genes in our dataset. Specifically, we queried the database with the 132 gene identifiers to retrieve all known protein-protein interactions among those gene products and their first neighbors. Network statistics were calculated using the NetworkAnalyzer plugin [31] in Cytoscape.

## Results

### Gene Selection

We have analyzed publicly available resequencing data for 132 genes involved in antibacterial innate immunity in individuals of African and European ancestry (see Methods). 129 genes are autosomal and only three genes (*IRAK1, TLR7 *and *TLR8*) are located in the X chromosome. All these genes have an unequivocal role in host defense against bacteria, with some of them also related to immune response against other pathogens, and were classified according to their main function in this system into five categories: receptors, adaptors, modulators, cytokines and effector molecules (Figure 1). We have also defined subclasses in the case of the pattern-recognition receptors (TLR2, TLR4, TLR5, NOD1, NOD2, TLR3 and TLR7-9 modules), which can be differentiated according to their location in the cell membrane (the first three), in the cytoplasm (NOD1 and NOD2), or in endosomes (TLR3, TLR7-9). Cytokines have been subclassified according to their involvement in different immune processes (acute phase, cellular immunity, anti-inflammatory cytokines, neutrophil function, chemokines, and defense against extracellular bacterial parasites). Several genes were included in a certain cytokine functional module due to their crucial role for the induction of the respective cytokine (e.g. *STAT3 *gene was included in the Th1 module) or for the role played in the function of the respective cytokine (e.g. *JAK3 *in the Th1 module). Five genes were not included in any of the functional classes defined (Additional file 1, Table S1).

### Nucleotide diversity within a network approach

Nucleotide diversity levels can provide a measure of the degree of conservation of the different genes and their comparison across functional classes may shed light on different intensities of purifying selection. We estimated the nucleotide diversity measured as the average number of differences between pairs of sequences (π) for the 132 genes within each population, and analyzed this information in the context of the position of the genes in the functional network and through the comparison of diversity levels among the five functional categories defined (see above).

We obtained the network for all the genes included in the study considering all known interactions for the 132 gene products, obtained from the MiMI database for molecular interactions (see Materials & Methods). Interactions could be retrieved for 126 of the 132 genes, and the final network consisted of 1,561 proteins with a total of 13,002 interactions. A finding emerging from previous studies on the influence of network topology on evolutionary rate is the observation that highly connected proteins in the protein-protein interaction network ("hubs") are more constrained in their evolution in respect to peripheral ones [32]. Furthermore, proteins in the periphery of the interactome have been shown to be more likely to be under positive selection in the human lineage than more centrally located ones [17]. In order to test whether this trend can also be observed among the innate immunity proteins in our dataset, we investigated the correlations between nucleotide diversity and two measures of the centrality of nodes within a network: the degree centrality (defined as the total number of links incident upon a node) and the betweenness centrality (defined as the fraction of all shortest paths that pass through a node). For these analyses, we considered all known interactions within the entire interactome for each of our 126 genes present in the MiMI database (see Methods), as they more accurately reflect a particular protein's position in the network than merely considering all interactions among themselves only.

Significant negative correlation values were obtained for degree centrality with nucleotide diversity in Africans (τ = -0.2, P = 0.001) and Europeans (τ = -0.19, P = 0.002) (Additional file 1, Figure S2). For betweenness centrality and nucleotide diversity the correlations were also negative and marginally significant (τ = -0.12, P = 0.05 for Africans; τ = -0.11, P = 0.08 for Europeans). When only the coding sequence is considered these correlations remain significant only in the case of degree centrality in Africans (τ = -0.15, P = 0.015) (Additional file 1, Figure S3), probably as a result of lack of power given the much smaller number of segregating sites. We repeated these analyses comparing both centrality indexes to divergence, measured as the ratio between nonsynonymous and synonymous changes (dN/dS) between humans and chimpanzees for each gene. Again, the correlations are negative and significant for degree centrality (τ = -0.16, P = 0.01) and marginally significant for betweenness centrality (τ = -0.12, P = 0.05) (Additional file 1, Figure S4), which is consistent with previous results, even though we are restricting our analyses to much fewer genes.

An alternative approach to the network analysis can be applied by considering the hierarchical structure of innate immunity signaling from a functional perspective. Figure 1 shows a "bow-tie" shaped organization in terms of the number of components and the flow of information: a relatively large number of receptor molecules recognizing different classes of pathogens all signal to a limited number of intracellular adaptor proteins which, in turn, can interact with proteins that act as modulators, and subsequently signal to a diverse array of downstream molecules, including cytokines and other effector molecules (Figure 1). This bow-tie structure characterized by a large number of "inputs", a relatively small number of central control nodes which elaborates the information, and a large number of "outputs", seems to be a topological organization widely adopted by metabolic networks [33-35] and also by the immunity system signaling network [36].

Given this structure, one can hypothesize that the amount of gene variation should follow a similar pattern to the one obtained in the context of the position of the genes in the network, with the adaptors common to all different pathogen responses being more constrained than both up- and downstream proteins, as previously reported [33]. Table 1 shows the average value of nucleotide diversity observed in the different functional classes, both using the whole sequence and only the coding sequence. Adaptors and modulators show significantly lower diversity values. When the whole available sequence for the gene is considered, adaptors show lower values of nucleotide diversity that are significant in Africans (unpaired t-test P = 0.01) although not in Europeans (P = 0.09). Considering only the coding sequence, this difference is significant in Africans for the adaptors (P = 0.006) and the modulators (P = 0.05). We also used a permutation procedure to test this hypothesis, where the mean nucleotide diversity calculated for each defined functional category in the original data is compared to 10,000 replicates with class labels randomly permuted. Significance is reached in adaptors for Africans (P = 0.004) and Europeans (P = 0.03) when using the whole sequence, and for adaptors in Africans for the coding sequence (P = 0.04) (Additional file 1, Figures S5 and S6). We repeated this analysis for molecular divergence and, although the adaptors and modulators also show the lowest dN/dS values among the five main functional categories, these differences were not statistically significant (Additional file 1, Table S2).

**Table 1:** Nucleotide diversity in the different functional classes

Category	N	π AFR^a^ (× 10 ^-4^)	π AFR CDS^b^ (× 10 ^-4^)	π EUR^a^ (× 10 ^-4^)	π EUR CDS^b^ (× 10 ^-4^)
Receptors	19	9.3	7.1	6.1	4.8
Adaptors	10	5.7	3.0	4.8	3.2
Modulators	6	7.9	3.2	6.3	3.4
Cytokines	69	8.8	5.8	7.2	4.7
Acute Phase	20	9.8	6.8	8.6	5.9
Cellular Immunity	10	8.6	4.4	7.0	3.3
Antiinflammatory Cytokines	10	8.1	4.1	6.3	4.2
Neutrophil	9	7.5	4.4	6.2	4.3
Chemokines	14	8.1	6.6	6.4	4.9
Extracellular	6	10.0	7.5	7.6	3.7
Effector	23	9.2	6.1	7.1	4.0
TOTAL	127	8.6	5.6	6.7	4.3

^a ^Whole sequence. ^b ^Coding sequence.

π, nucleotide diversity (average of pairwise differences).

N, number of genes included in each category.

### Some functional classes are preferential targets for adaptive selection

**Figure 2:**
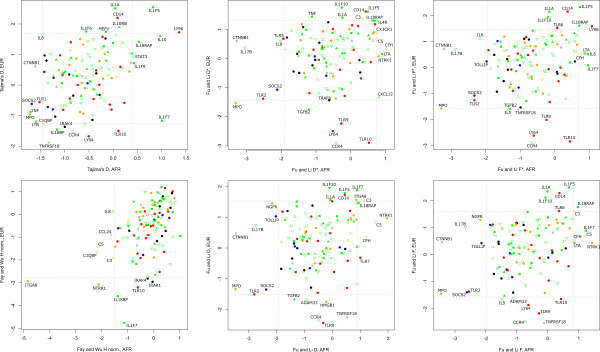
**Neutrality tests results**. Scatter plots for the six neutrality tests performed. Genes in the same functional class are represented in the same colors as defined in Figure 1. Cytokines are in green and the subcategories are represented by different symbols (filled square for acute phase, filled triangle for antiinflamatory cytokines, empty circle for cellular immunity, empty square for chemokynes, empty triangle for extracellular, empty inverted triangle for neutrophil). For the effector molecules, an empty diamond represents the complement category.

We evaluated if some functional categories are also preferential targets for positive or balancing selection. We performed six different neutrality tests (see Methods) to detect genes with an excess of rare or intermediate variants, which after correcting for demographiy can suggest positive or balancing selection. Tajima's D and Fu and Li's D* and F* use the intraspecific variation data only, whereas Fu and Li's D and F and the normalized Fay and Wu's H tests compare these intraspecific information to a sequence from another species (outgroup). Tajima's D, and Fu and Li's D* and F* tests are based on the comparison of low frequency variants (Tajima's D) or singletons (Fu and Li's D* and F*) to intermediate variants. Fu and Li D and F tests are based on the comparison of mutations in the external branches of the genealogy to the total number of mutations and to the average number of nucleotide differences between pairs of sequences, respectively. In these five tests, values lower than expected under neutrality are obtained when there is an excess of rare variants (low frequency, singletons, mutations at external branches), and values higher than expected under neutrality are obtained when there is an excess of intermediate frequency variants. Fay and Wu's H and its normalized test have been proven to have power to detect the selective sweeps increasing the frequency of the derived alleles (those different to the allele in the outgroup species) produced by positive selection or initial stages of balancing selection [25]. The significance of these tests was evaluated by comparing the results for each gene to the rest of the genes included in the study (Figure 2), since all the sequences are supposed to share the same demographic history. We considered a gene to show evidence of positive or balancing selection if it was included in the 95^th ^upper or lower percentiles for two or more neutrality test in the same population. Additionally, for those genes statistical significance was also assessed by means of coalescent simulations that resemble the demographic history of Africans and Europeans (see Methods) (Additional file 1, Table S3). These methods should exclude the possibility of these signals being produced randomly or by demographic events such as expansion or population contractions. Moreover, our aim is to identify different selective pressures acting on the different functional classes, rather than describing unequivocal signatures of selection in a particular gene. These possible random effects of demography in a particular gene are also minimized by pooling together the genes in the same functional class, as we did for the subsequent analysis.

Table 2 shows the list of candidate genes for positive and balancing selection in each population. Coalescent simulations corroborated the results in all cases, with all the genes showing significant results in at least two of the neutrality tests (Additional file 1, Table S3). Although the neutrality tests used have more power to detect positive rather than purifying selection [37] we have indicated among the genes under positive selection those with significant results in the Fay and Wu's H test as genes with unequivocal signatures of positive selection (Table 2). From the total of 132 genes, 31 show signs of adaptive selection: 17 of positive selection (8 in Africans and 10 in Europeans, with *MPO *in the two populations) and 14 of balancing selection (9 in Africans and 10 in Europeans, with *LY96, IL18RAP, IL1F5, IL1F7 *and *C3 *in the two populations). Some of them have been previously reported by independent studies using the same or similar datasets: balancing selection in *IL1F5, IL1F7, IL1F10 *and *IL18RAP *[13] and positive selection in the *TLR1*-*TLR6*-*TLR10 *region finally attributed to *TLR1 *[38]. It has to be noted that *IL1A, IL1F7, IL1F5 *and *IL1F10 *are located in a region spanning 300 kb at chromosome 2. As discussed in Fumagalli et al [13], it seems improbable that a hitchhiking effect is producing all these signatures even between the closest genes (*IL1F5 *and *IL1F10*), given the low LD and presence of recombination hotspots in this region, as well as the fact that balancing selection signatures have been proven to spread only over small regions. Moreover, other genes included in this region (*IL1B, IL1F9, IL1F6 *and *IL1F8*) did not show excess of intermediate variants.

**Table 2:** Number of genes in each functional class with evidences of adaptive selection

Functional Class		Africans		Europeans	
	**N**	**Positive Selection**	**Balancing Selection**	**Positive Selection**	**Balancing Selection**

Receptors	19	*TLR2*	*LY96*	*LY64*^a^, *TLR9, TLR10*^a^	*CD14, LY96, TLR6*
Adaptors	10	-	-	*IRAK4*^a^	-
Modulators	6	*SOCS2*^a^, *TOLLIP*	-	-	-
Acute Phase	20	-	*LTA, IL18RAP, IL1F5, IL1F7*	*IL18BP*^a^	*IL1F5, IL1A, IL1F10, IL18RAP, IL1F7*
Cellular Immunity	10	-	-	-	-
Antiinf. Cytokines	10	-	-	*TGFB2*	-
Neutrophil	9	*IL17B*^a^	-	-	-
Chemokines	14	*IL8*^a^	-	*CCR4*	-
Extracellular	6	-	-	*ADAM33*	-
Complement	8	-	*CFH, C5, C3*	-	*C3*
Effector	15	*MPO, NGFR*	*NTRK1*	*MPO*	*ITGA8*
Non classified	5	*CTNNB1*	-	*TNFRSF18*	-

N, number of genes in each functional category.

^a ^Genes with evidences of positive selection showing an excess of derived alleles at high frequencies (Fay and Wu's H test).

Beyond signatures of adaptive selection on specific genes, we have analyzed the effect of functional classes. Figure 2 shows the results of the six neutrality tests performed. We tested for an excess of candidate genes under positive or balancing selection (or both) in each functional class, by comparing the number of genes with and without significant evidence of adaptive selection in this class against the rest of the genes included in the study (see Materials and Methods). Genes with statistical evidence for positive or balancing selection are not randomly distributed among the different functional classes, and tend to accumulate in a reduced number of classes. Specifically, there is an unexpected high number of acute phase proteins showing signatures of balancing selection, both in Africans and Europeans (Fisher's exact test P = 0.03 and 0.008, respectively). This excess of genes under balancing selection in this functional category remains statistically significant even if *IL1F5 *and *IL1F10 *signatures in Europeans are considered to be produced by the same selective event (P = 0.03). Remarkably, all genes in that category with signatures of balancing selection in both Europeans and Africans are included in the IL1 or TNF family (Figure 1). Moreover *MEFV *also shows an excess of intermediate variants in one of the neutrality tests in Europeans, and signatures of balancing selection have been previously reported [12,13]. The complement proteins also show a significant enrichment for genes under balancing selection in Africans (Fisher's exact test P = 0.01). Finally, the receptors are also overrepresented among the genes showing signatures of positive or balancing selection, suggesting adaptive selection events. In this case, this tendency is only observed in Europeans (Fisher's exact test P = 0.04), and is due to an excess of extracellular receptors (Fisher's exact test P = 0.02).

## Discussion

A high number of genes analyzed here have showed signs of adaptive selection. Even if data is not comparable to whole genome scans looking at the footprint of positive selection with SNP data, the five studies published so far [2-4,6,39] all reviewed in [40] find from 444 to 1,030 genomic regions as candidates for positive selection, which represents a very small fraction of the genome (< 4%). Thus our results support previous evidence that immunity genes have likely been preferential targets of recent adaptive selection in human populations. The excess of genes under adaptive selection is still more striking when balancing selection is considered. Balancing selection has traditionally been considered to have acted less frequently on human populations, and the number of reported cases is smaller in comparison to positive selection. However, this difference could be at least partially due to the intrinsic difficulties presented by its detection [1].

Scientific literature contains several examples of host-pathogen interaction genes with evidences of balancing selection. In addition to some well known cases as the human major histocompatibility complex, more recently several works have described the action of balancing selection on additional immunity genes [10-14,41-43] and blood group antigen genes that are supposed to act as molecular targets for pathogens [12,13,43-45]. The first genome scan for signatures of balancing selection has also confirmed an enrichment of immune system and host-pathogen interaction related genes [1].

**Figure 3:**
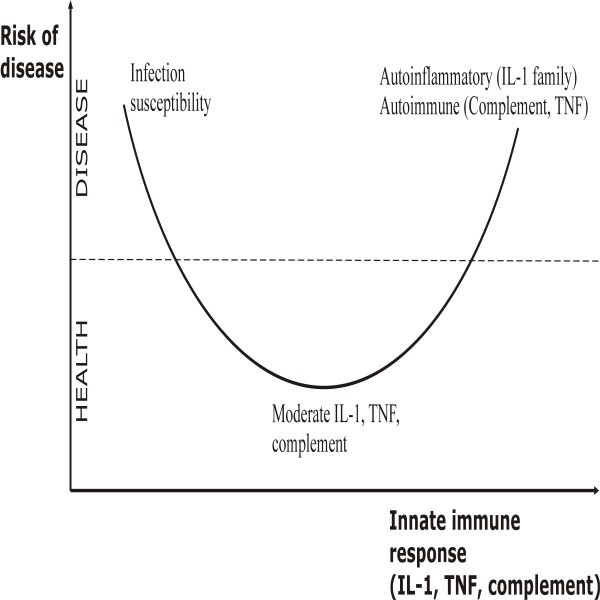
**Host defense versus autoimmune and autoinflammatory diseases**. Model representing the counterbalanced effect of host defense against pathogens on the one hand, and the deleterious effects of an exaggerated immune response in autoimmune and autoinflammatory disease on the other hand.

The high number of immunity genes that are under balancing selection is presumably due to a beneficial effect in infections, that is counterbalanced by deleterious effects in autoinflammatory and autoimmune conditions (Figure 3) [11]. The IL-1 family is overrepresented (most abundantly and strongly under balanced selection, with 6 genes represented in both European and African populations). Additionally, signatures of balancing selection have also been reported in the Mediterranean Fever gene, *MEFV*, another member of this family [12,13]. There are also members of the complement (three genes) and the TNF family (TNFβ/LTα) showing signatures of balancing selection. While their role in host defense is well documented, these genes encode the main proinflammatory mediators of the two most important classes of sterile inflammation disorders: the autoinflammatory diseases (e.g. Crohn's disease, gout, autoinflammatory syndromes, Behcet's disease) in which inflammation is mediated by the IL-1 family, and the autoimmune diseases (e.g. rheumatoid arthritis, systemic lupus erythematous, type 1 diabetes) in which the TNF family and complement play a central role [46]. Alternatively, variants found at genes under balancing selection could be conferring susceptibility to different pathogens and their effect on autoimmunity would be a consequence of past humans adaptations, as suggested recently for IFIH1, a innate immunity gene involved in resistance to virus [15]. The fact that some of the genes found to be under balancing selection are exactly those encoding the major mediators of human autoinflammatory and autoimmune pathologies is relevant.

The dissection of the innate immune system presented here allows inferring other important biological consequences. Selection signatures at receptor genes support the major evolutionary flexibility described in extracellular TLRs compared to the intracellular ones [38,47]. One important question that may be also asked is which major class of bacteria could have exerted the evolutionary pressures observed here. As the innate immune system is relatively non-specific, any genetic information obtained in the present study cannot refer to specific species or even families, but rather to broad groups of pathogens: both Gram-positive bacteria (e.g. *Staphylococci *and *Streptococci*, mycobacteria) and Gram-negative bacteria (*Salmonella *and other enterobacteriaceae, *Yersinia pestis*) contain pathogens that had a major impact on human populations during history. An answer to the question of whether both these two groups of bacteria exerted evolutionary pressures cannot be obtained from the selection profile of effector genes such as defensins or cytokines, as these proteins are generally necessary for host defense to both Gram-positive and Gram-negative bacteria. In contrast, a certain level of specificity of the innate immune response can be seen by the recognition of either Gram-positive bacteria mainly by the TLR2/TLR1/TLR6/TLR10 cluster, while Gram-negative bacteria recognition is heavily reliant on the TLR4 pathway [48]. In our study we found signatures of selection in genes from both the TLR2 cluster (*TLR2, TLR6, TLR10*) and the TLR4 pathway (*LY64, LY96, CD14*), suggesting that both Gram-negative and Gram-positive bacteria have exerted pressure on innate immunity genes. In addition, TLR9, a pathogen recognition receptor recognizing unmethylated DNA from both Gram-negative and Gram-positive bacteria [49] showed also signatures of selection in Europeans. The exclusive positive selection of *TLR9 *and *TLR10 *(or TLR1) pathways in Europeans may be related to more recent responses that could have been related to the introduction of agriculture and zoonotic diseases (e.g. *Brucella, Coxiella) *or the major epidemics of the medieval periods (e.g. *Yersinia pestis *causing pandemics including pneumonic, septicaemic and bubonic plague). However, at this point one also has to be aware that some of these receptors have also a role for recognition of other types of pathogens (e.g. TLR9 for recognition of DNA from certain viruses or parasites, or TLR2/TLR4 recognizing also malaria and fungal structures) and although these are secondary recognition pathways for these microorganisms, one cannot exclude evolutionary pressures of non-bacterial pathogens on some of the genes analyzed here.

## Conclusions

Overall, our results suggest that the innate immune system has had enough plasticity to play an important role in the adaptation of modern humans to new environments. Analysis of the protein-protein interaction network suggests a structural and functional constraint on genes that occupy a central position in the innate immunity network. This network is organized into a bow tie structure, consisting in a large number of receptors, a central small number of adaptor genes (the bow tie knot) and a large number of effectors. The bow tie structure is already known to characterize many metabolic and system networks and it is recognized as a topological feature which provides robustness to systems [33,50]. It also has the advantage of making robustness compatible with a high capacity to evolve (or evolvability): the knot provides robustness to the system by easily accommodating for perturbations, while a great variability is allowed at the edges of the bow tie making possible the generation of new functionalities [33]. While the core of the bow tie structure is highly conserved, signatures of adaptation are found at the bow tie edges. Therefore, the amount of selection acting on each gene appeared therefore highly dependent on its position in the network structure. This clearly shows the strength of the constraint represented by the topology of the network on the evolution of individual genes, which is able to explain much better the adaptive landscape of innate immunity in humans.

## Authors' contributions

FCas and JB designed the study. AM and MGN designed the functional network categories. FCas, MS, HL and LM performed the analyses. RL assisted the processing of sequencing data. FCas, MS, HL, LM, RL, FCal, PA and JB analyzed the data. FCas, MS, AM, MGN and JB elaborated the discussion. FCas, MS, MGN and JB wrote the manuscript with important contributions from AM. All the authors participated in critically reviewing the paper and approved the final version of the manuscript.

## Supplementary Material

Additional file 1 **Additional Figures and Tables**. Additional figures and tables containing the comparison of nucleotide diversity in Africans and Europeans (Figure S1), the correlation between nucleotide diversity for the whole sequence and the degree and betweenness centrality (Figure S2), the correlation between nucleotide diversity for the coding sequence and the degree and betweenness centrality (Figure S3), the correlation between divergence and the degree and betweenness centrality (Figure S4), the comparison of the mean nucleotide diversity in each functional class for the whole sequence to the empirical distributions obtained from 10,000 permutations (Figure S5), the comparison of the mean nucleotide diversity in each functional class for the coding sequence to the empirical distributions obtained from 10,000 permutations (Figure S6), a list of the genes included in the study and their functional classification (Table S1), the mean divergence values in the different functional classes (TableS2), and the significance of the neutrality tests estimated by using coalescent simulations (Table S3). Click here for file
