# Supplementary material for: Genetic adaptation of the antibacterial human innate immunity network
Source: BMC Evol Biol. 2011 Jul 11;11:202. doi: 10.1186/1471-2148-11-202 (PMC3155920; doi:10.1186/1471-2148-11-202)
Supplement: Additional file 1 — Additional Figures and Tables. Additional figures and tables containing the comparison of nucleotide diversity in Africans and Europeans (Figure S1), the correlation between nucleotide diversity for the whole sequence and the degree and betweenness centrality (Figure S2), the correlation between nucleotide diversity for the coding sequence and the degree and betweenness centrality (Figure S3), the correlation between divergence and the degree and betweenness centrality (Figure S4), the comparison of the mean nucleotide diversity in each functional class for the whole sequence to the empirical distributions obtained from 10,000 permutations (Figure S5), the comparison of the mean nucleotide diversity in each functional class for the coding sequence to the empirical distributions obtained from 10,000 permutations (Figure S6), a list of the genes included in the study and their functional classification (Table S1), the mean divergence values in the different functional classes (TableS2), and the significance of the neutrality tests estimated by using coalescent simulations (Table S3). [file 1471-2148-11-202-S1.DOC]

Figure S1. Nucleotide diversity in Africans and Europeans.

Nucleotide diversity in the different genes and functional classes in African and European individuals. Colors for each functional class are the same than in Figure 1. As expected and in agreement with previous reports, nucleotide diversity levels are higher in Africans than in Europeans (paired t-test P < 0.001) (Cavalli-Sforza and Feldman 2003). A, Nucleotide diversity for the whole sequence; B, Nucloetide diversity for the coding sequence.

A

B

Figure S2. Nucleotide diversity vs degree and betweenness centrality (whole sequence).

Scatterplots of nucleotide diversity (whole sequence) versus degree and betweenness centrality for each protein in both populations. Colors for each functional class are the same than in Figure 1. Kendall’s tau as well as significance of the correlations are indicated. A, Africans; B, Europeans.


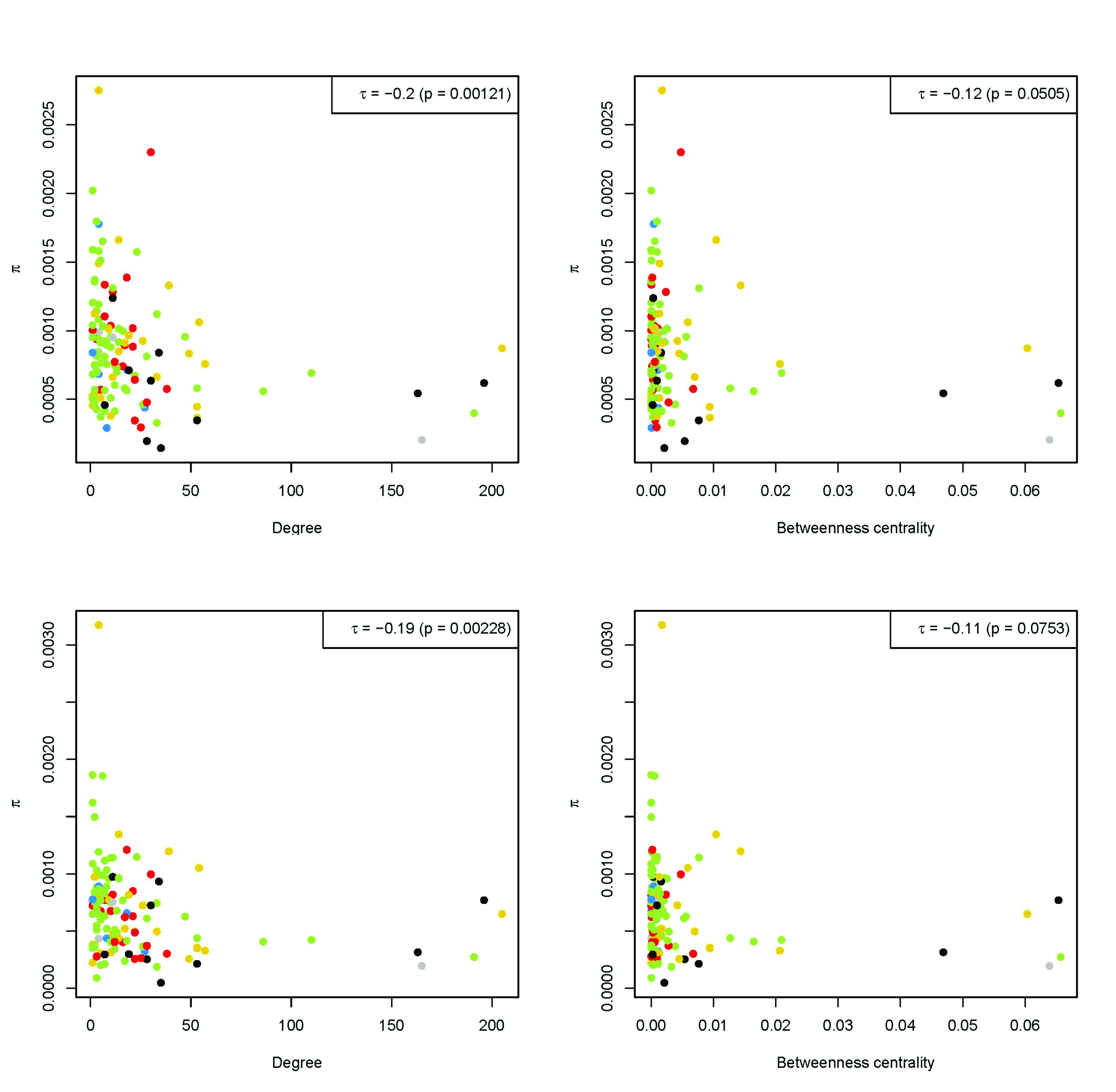


Figure S3. Nucleotide diversity vs degree and betweenness centrality (coding sequence).

Scatterplots of nucleotide diversity (coding sequence) versus degree and betweenness centrality for each protein in both populations. Colors for each functional class are the same than in Figure 1. Kendall’s tau as well as significance of the correlations are indicated. A, Africans; B, Europeans.


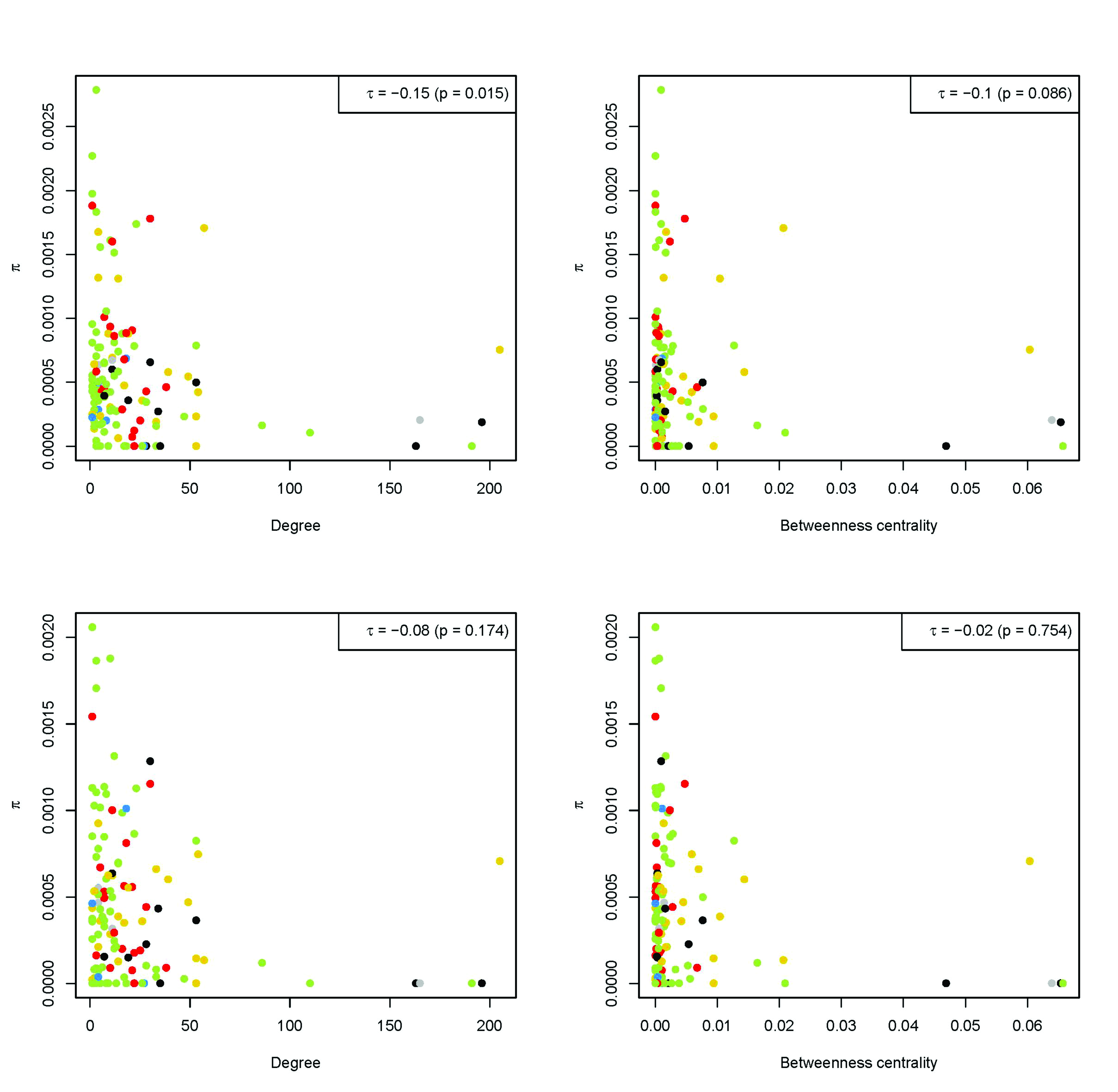


Figure S4. Divergence vs degree and betweenness centrality.

Scatterplots of divergence versus betweenness centrality and degree for each protein (123 genes, after exluding the genes with dS = 0). Colors for each functional class are the same than in Figure 1. Kendall’s tau as well as significance of the correlations are indicated.


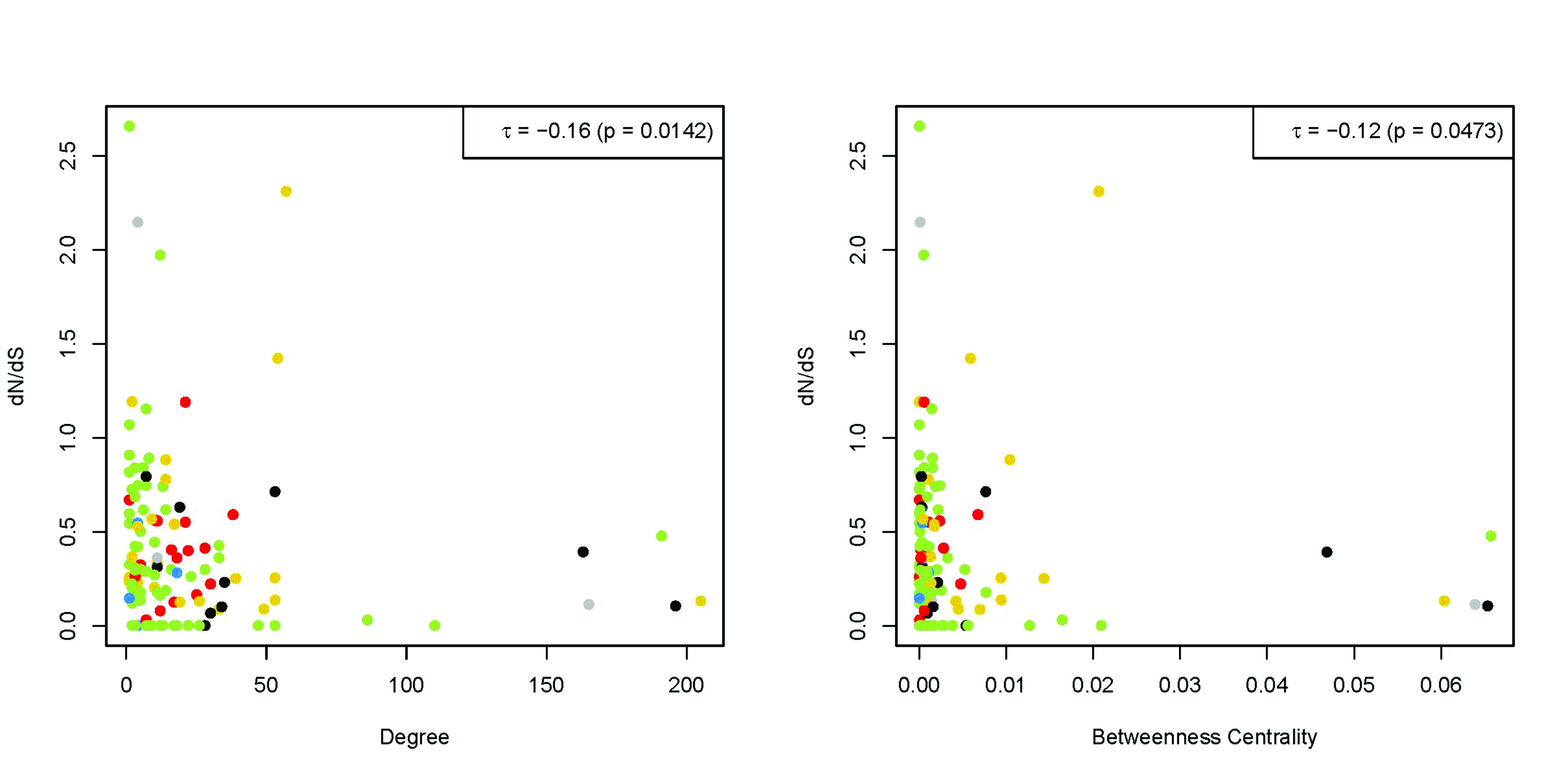


**Figure S5. Empirical distributions of nucleotide diversity from 10,000 permutations (whole sequence).**

Empirical distributions of mean nucleotide diversity (whole sequence) for each level of signalling hierarchy, obtained from 10,000 permutations. Red line indicates observed value for each population in each level. Levels are: 1 – Receptors; 2 – Adaptors; 3 – Modulators; 4 – Cytokines; 5 – Effectors.


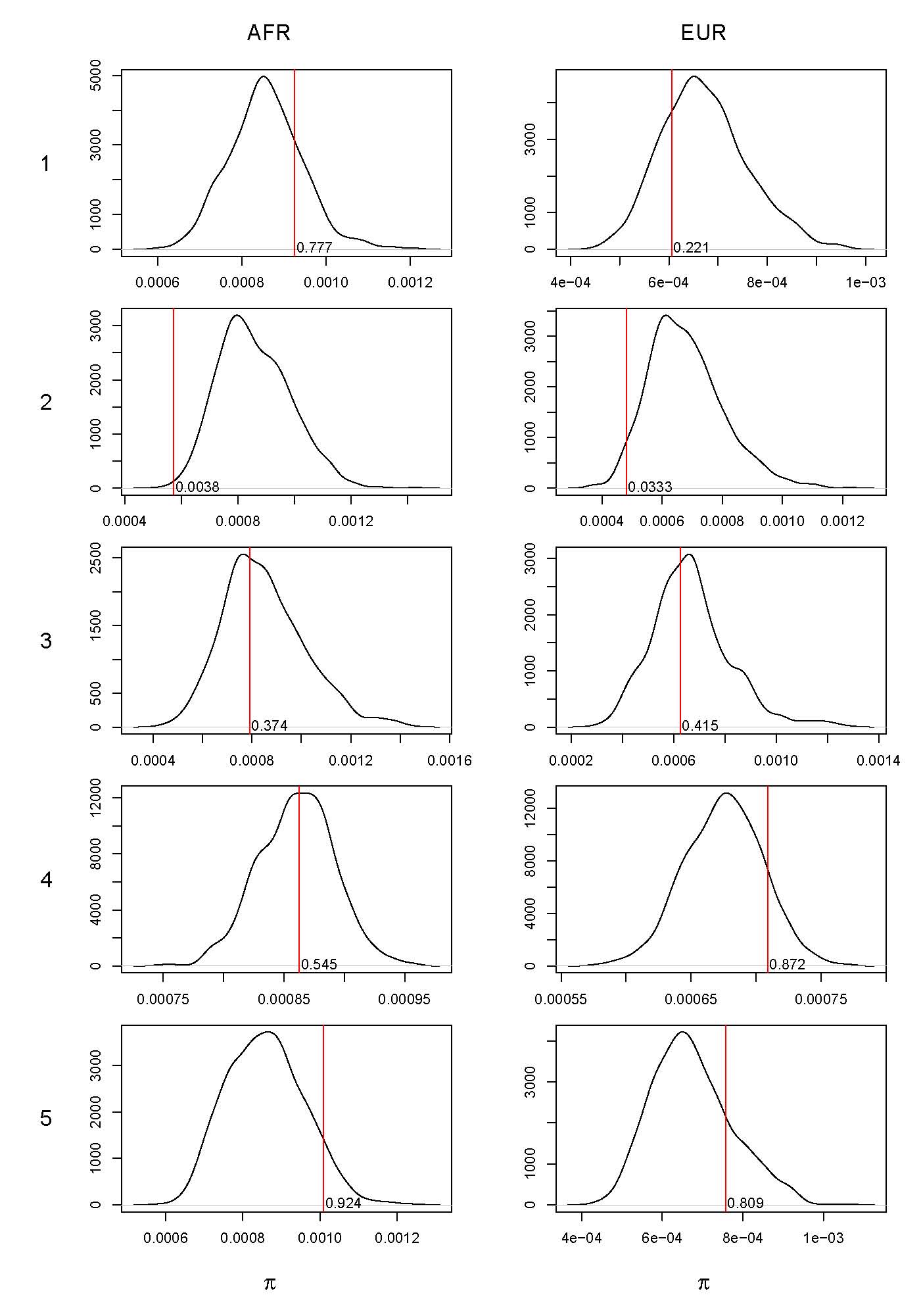


**Figure S6. Empirical distributions of nucleotide diversity from 10,000 permutations (coding sequence).**

Empirical distributions of mean nucleotide diversity (coding sequence) for each level of signalling hierarchy, obtained from 10,000 permutations. Red line indicates observed value for each population in each level. Levels are: 1 – Receptors; 2 – Adaptors; 3 – Modulators; 4 – Cytokines; 5 – Effectors.


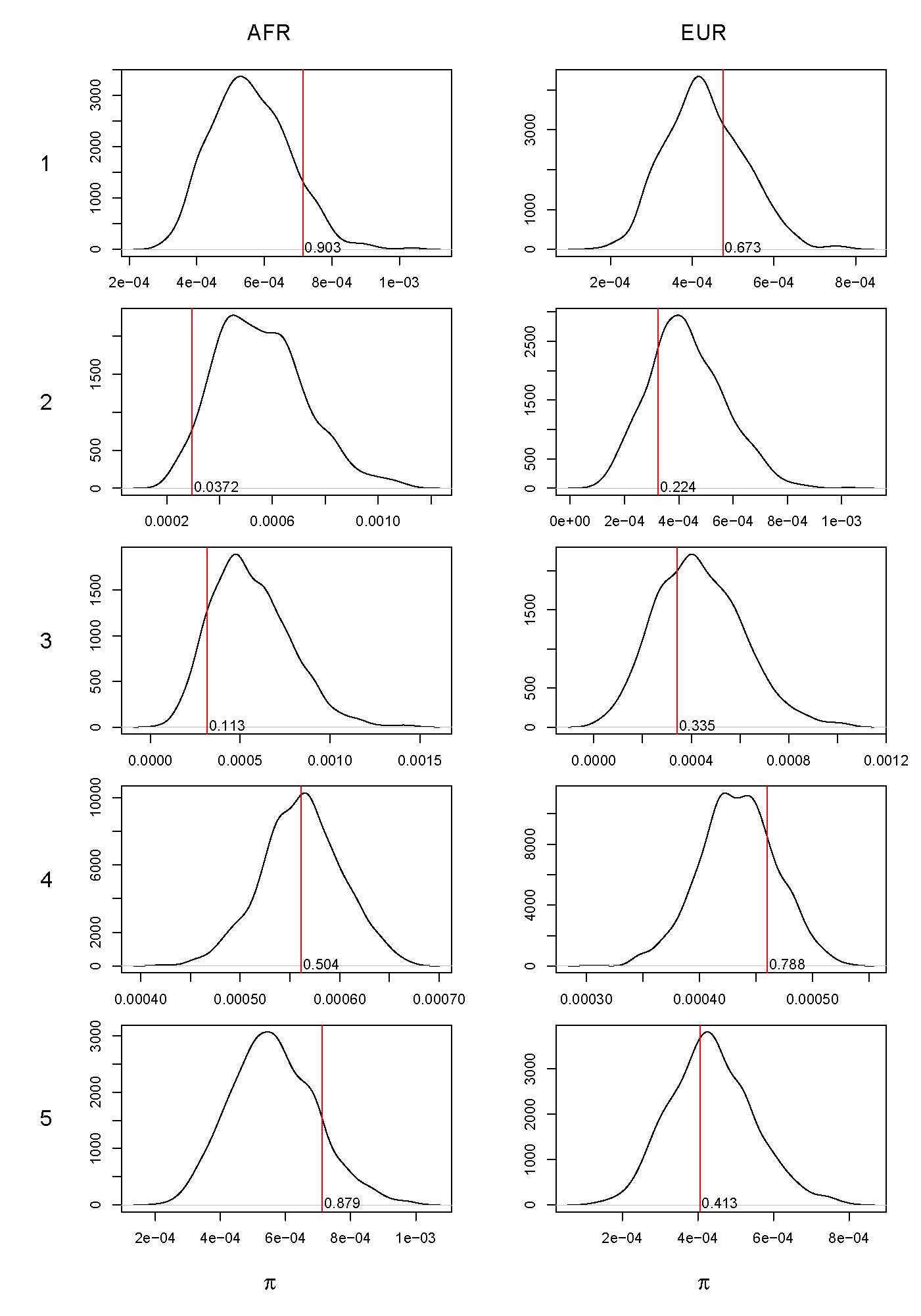


**Table S1. Genes included in the study and functional classification.**

| **Gen** | **FunctionA** | **FunctionB** |
| --- | --- | --- |
| *IRAK1* | Adaptor | Adaptor |
| *IRAK2* | Adaptor | Adaptor |
| *IRAK3* | Adaptor | Adaptor |
| *IRAK4* | Adaptor | Adaptor |
| *MYD88* | Adaptor | Adaptor |
| *TIRAP* | Adaptor | Adaptor |
| *TRAF2* | Adaptor | Adaptor |
| *TRAF4* | Adaptor | Adaptor |
| *TRAF6* | Adaptor | Adaptor |
| *TRIF* | Adaptor | Adaptor |
| *IL18* | Cytokines | Acute Phase |
| *IL18BP* | Cytokines | Acute Phase |
| *IL18R1* | Cytokines | Acute Phase |
| *IL18RAP* | Cytokines | Acute Phase |
| *IL1A* | Cytokines | Acute Phase |
| *IL1B* | Cytokines | Acute Phase |
| *IL1F10* | Cytokines | Acute Phase |
| *IL1F5* | Cytokines | Acute Phase |
| *IL1F6* | Cytokines | Acute Phase |
| *IL1F7* | Cytokines | Acute Phase |
| *IL1F8* | Cytokines | Acute Phase |
| *IL1F9* | Cytokines | Acute Phase |
| *IL1R1* | Cytokines | Acute Phase |
| *IL1R2* | Cytokines | Acute Phase |
| *IL6* | Cytokines | Acute Phase |
| *LTA* | Cytokines | Acute Phase |
| *LTB* | Cytokines | Acute Phase |
| *LTBR* | Cytokines | Acute Phase |
| *MEFV* | Cytokines | Acute Phase |
| *TNF* | Cytokines | Acute Phase |
| *IL10* | Cytokines | Antiinflamatory Cytokines |
| *IL10RA* | Cytokines | Antiinflamatory Cytokines |
| *IL10RB* | Cytokines | Antiinflamatory Cytokines |
| *TGFA* | Cytokines | Antiinflamatory Cytokines |
| *TGFB1* | Cytokines | Antiinflamatory Cytokines |
| *TGFB2* | Cytokines | Antiinflamatory Cytokines |
| *TGFB3* | Cytokines | Antiinflamatory Cytokines |
| *TGFBR1* | Cytokines | Antiinflamatory Cytokines |
| *TGFBR2* | Cytokines | Antiinflamatory Cytokines |
| *TGFBR3* | Cytokines | Antiinflamatory Cytokines |
| *IFNA1* | Cytokines | Cellular Immunity |
| *IFNAR1* | Cytokines | Cellular Immunity |
| *IFNAR2* | Cytokines | Cellular Immunity |
| *IL12A* | Cytokines | Cellular Immunity |
| *IL12B* | Cytokines | Cellular Immunity |
| *IL12RB1* | Cytokines | Cellular Immunity |
| *IL12RB2* | Cytokines | Cellular Immunity |
| *IL17* | Cytokines | Cellular Immunity |
| *JAK3* | Cytokines | Cellular Immunity |
| *STAT1* | Cytokines | Cellular Immunity |
| *STAT3* | Cytokines | Cellular Immunity |
| *CCL11* | Cytokines | Chemokines |
| *CCL2* | Cytokines | Chemokines |
| *CCL24* | Cytokines | Chemokines |
| *CCL26* | Cytokines | Chemokines |
| *CCR2* | Cytokines | Chemokines |
| *CCR4* | Cytokines | Chemokines |
| *CCR6* | Cytokines | Chemokines |
| *CX3CR1* | Cytokines | Chemokines |
| *CXCL12* | Cytokines | Chemokines |
| *CXCR4* | Cytokines | Chemokines |
| *CXCR6* | Cytokines | Chemokines |
| *IL8* | Cytokines | Chemokines |
| *IL8RA* | Cytokines | Chemokines |
| *IL8RB* | Cytokines | Chemokines |
| *C1QA* | Effector | Complement |
| *C1QBP* | Effector | Complement |
| *C2* | Effector | Complement |
| *C3* | Effector | Complement |
| *C3AR1* | Effector | Complement |
| *C4BPB* | Effector | Complement |
| *C5* | Effector | Complement |
| *CFH* | Effector | Complement |
| *ADAM33* | Cytokines | Extracellular |
| *IL4* | Cytokines | Extracellular |
| *IL4R* | Cytokines | Extracellular |
| *IL5* | Cytokines | Extracellular |
| *IL5RA* | Cytokines | Extracellular |
| *PTGDR* | Cytokines | Extracellular |
| *IL17B* | Cytokines | Neutrophil |
| *IL17D* | Cytokines | Neutrophil |
| *IL17E* | Cytokines | Neutrophil |
| *IL17F* | Cytokines | Neutrophil |
| *IL17RB* | Cytokines | Neutrophil |
| *IL21* | Cytokines | Neutrophil |
| *IL21R* | Cytokines | Neutrophil |
| *IL23R* | Cytokines | Neutrophil |
| *ITGA2* | Effector | Adhesion |
| *ITGA8* | Effector | Adhesion |
| *ITGAL* | Effector | Adhesion |
| *CLCA1* | Effector | Effector |
| *CRP* | Effector | Effector |
| *DEFB1* | Effector | Effector |
| *EGF* | Effector | Effector |
| *EGFR* | Effector | Effector |
| *HMGB1* | Effector | Effector |
| *MPO* | Effector | Effector |
| *MUC2* | Effector | Effector |
| *MUC7* | Effector | Effector |
| *NGFR* | Effector | Effector |
| *NOS1* | Effector | Effector |
| *NTRK1* | Effector | Effector |
| *CD209* | Modulators | Modulators |
| *SOCS2* | Modulators | Modulators |
| *SOCS3* | Modulators | Modulators |
| *SOCS4* | Modulators | Modulators |
| *SOCS5* | Modulators | Modulators |
| *TOLLIP* | Modulators | Modulators |
| *CTNNB1* | ND | ND |
| *DTR* | ND | ND |
| *GPR44* | ND | ND |
| *SCGB1A1* | ND | ND |
| *TNFRSF18* | ND | ND |
| *BPI* | Receptors | Receptors |
| *CARD11* | Receptors | Receptors |
| *CARD15 (NOD2)* | Receptors | Receptors |
| *CARD4 (NOD1)* | Receptors | Receptors |
| *CD14* | Receptors | Receptors |
| *LBP* | Receptors | Receptors |
| *LY64* | Receptors | Receptors |
| *LY86* | Receptors | Receptors |
| *LY96* | Receptors | Receptors |
| *TLR1* | Receptors | Receptors |
| *TLR2* | Receptors | Receptors |
| *TLR3* | Receptors | Receptors |
| *TLR4* | Receptors | Receptors |
| *TLR5* | Receptors | Receptors |
| *TLR6* | Receptors | Receptors |
| *TLR7* | Receptors | Receptors |
| *TLR8* | Receptors | Receptors |
| *TLR9* | Receptors | Receptors |
| *TLR10* | Receptors | Receptors |

**Table S2**. **Divergence (dN / dS) in the different functional classes.**

| Category | N | dN / dS |
| --- | --- | --- |
| Receptors | 17 | 0.41 |
| Adaptor | 10 | 0.33 |
| Modulators | 4 | 0.24 |
| Cytokines | 65 | 0.41 |
| Acute Phase | 19 | 0.45 |
| Cellular Immunity | 10 | 0.27 |
| Antiinflamatory Cytokines | 10 | 0.19 |
| Neutrophil | 7 | 0.48 |
| Chemokines | 13 | 0.50 |
| Extracellular | 6 | 0.53 |
| Effector | 23 | 0.49 |
| TOTAL | 118 | 0.41 |

N, number of genes included in each category. The following genes are not included: *CCL26*, *IL17*, *IL17D*, *IL1F8*, *LY96*, *SOCS2*, *SOCS3*, *TLR5* (dS = 0).

**Table S3. Neutrality tests results and significance estimated by means of coalescence simulations.**

| **Gene** | **T(D)_AFR** | **P** | **FL(D*)_AFR** | **P** | **FL(F*)_AFR** | **P** | **FL(D)_AFR** | **P** | **FL(F)_AFR** | **P** | **FW(H)_AFR** | **P** | **FW(N)_AFR** | **P** |
| --- | --- | --- | --- | --- | --- | --- | --- | --- | --- | --- | --- | --- | --- | --- |
| C5 | 0.07 | < 0.001 | 0.75 | < 0.001 | 0.59 | < 0.001 | 1.55 | < 0.001 | 1.2 | < 0.001 | -39.71 | < 0.001 | -2.13 | < 0.001 |
| CFH | 0.01 | 0.003 | 1.02 | < 0.001 | 0.77 | < 0.001 | 1.21 | < 0.001 | 0.88 | < 0.001 | 1.64 | ns | 0.06 | ns |
| CTNNB1 | -1.61 | 0.019 | -3.28 | 0.002 | -3.21 | 0.002 | -3.45 | 0.005 | -3.42 | 0.005 | -1.35 | ns | -0.49 | ns |
| IL17B | -1.29 | 0.041 | -3.05 | 0.001 | -2.89 | 0.001 | -2.74 | 0.009 | -2.66 | 0.01 | -4.42 | 0.006 | -1.03 | 0.004 |
| IL18RAP | 0.54 | 0.001 | 0.62 | < 0.001 | 0.71 | < 0.001 | 0.95 | < 0.001 | 0.98 | < 0.001 | 4.58 | ns | 0.34 | ns |
| IL1F5 | 0.82 | 0.001 | 0.49 | 0.017 | 0.73 | 0.002 | 0.45 | 0.024 | 0.69 | 0.003 | -2.07 | ns | -0.25 | ns |
| IL1F7 | 1.01 | < 0.001 | 0.69 | 0.012 | 0.96 | 0.001 | 0.9 | 0.008 | 1.24 | < 0.001 | -8.03 | ns | -1.13 | ns |
| IL8 | -1.45 | 0.027 | -2.02 | 0.042 | -2.17 | 0.03 | -1.16 | ns | -1.54 | ns | -6.93 | <0.001 | -1.56 | 0.002 |
| LTA | 0.39 | 0.039 | 0.9 | 0.022 | 0.86 | 0.021 | 0.89 | 0.028 | 0.87 | 0.013 | 0.73 | ns | 0.26 | ns |
| LY96 | 1.36 | 0.009 | 0.7 | ns | 1.08 | 0.029 | -0.21 | ns | -0.29 | ns | 0.57 | ns | 0.44 | ns |
| MPO | -1.72 | < 0.001 | -3.31 | < 0.001 | -3.29 | < 0.001 | -3.46 | < 0.001 | -3.45 | < 0.001 | -2.68 | 0.049 | -0.37 | ns |
| NGFR | -0.9 | ns | -1.41 | ns | -1.46 | ns | -2.15 | 0.035 | -2.18 | 0.024 | 1.1 | ns | 0.17 | ns |
| NTRK1 | -0.61 | ns | 0.8 | < 0.001 | 0.33 | 0.004 | 1.96 | < 0.001 | 1.44 | < 0.001 | -13.2 | < 0.001 | -2.12 | < 0.001 |
| SOCS2 | -1.68 | 0.001 | -2.11 | 0.019 | -2.33 | 0.004 | -2.37 | 0.023 | -2.62 | 0.007 | -5.17 | 0.002 | -1.36 | < 0.001 |
| TLR2 | -0.89 | ns | -2.52 | 0.016 | -2.33 | 0.022 | -2.79 | 0.018 | -2.55 | 0.032 | 1.48 | ns | 0.56 | ns |
| TOLLIP | -1.35 | 0.002 | -1.83 | 0.011 | -1.98 | 0.005 | -2.02 | 0.026 | -2.14 | 0.009 | 0.49 | ns | 0.03 | ns |
|  |  |  |  |  |  |  |  |  |  |  |  |  |  |  |
| **Gene** | **T(D)_EUR** | **P** | **FL(D*)_EUR** | **P** | **FL(F*)_EUR** | **P** | **FL(D)_EUR** | **P** | **FL(F)_EUR** | **P** | **FW(H)_EUR** | **P** | **FW(N)_EUR** | **P** |
| ADAM33 | -0.5 | ns | -1.18 | 0.038 | -1.12 | 0.036 | -1.68 | 0.02 | -1.62 | 0.015 | -7.76 | 0.005 | -1.5 | 0.005 |
| CCR4 | -1.53 | 0.025 | -2.9 | 0.008 | -2.89 | 0.008 | -2.36 | 0.014 | -2.48 | 0.015 | -1.3 | ns | -0.89 | ns |
| CD14 | 2.2 | < 0.001 | 1.62 | 0.001 | 2.15 | < 0.001 | 1.73 | < 0.001 | 2.28 | < 0.001 | 0.05 | ns | 0.02 | ns |
| IL18BP | -1.46 | 0.019 | 0.24 | ns | -0.41 | ns | 0.22 | ns | -0.46 | ns | -10.91 | 0.001 | -3.59 | 0.001 |
| IL18RAP | 1.41 | 0.001 | 1.31 | < 0.001 | 1.61 | < 0.001 | 1.47 | < 0.001 | 1.77 | < 0.001 | 8.66 | 0.001 | 0.7 | 0.014 |
| IL1A | 2.51 | < 0.001 | 1.41 | 0.009 | 2.15 | < 0.001 | 1.57 | 0.006 | 2.35 | < 0.001 | 4 | ns | 0.61 | ns |
| IL1F10 | 1.38 | 0.001 | 1.75 | < 0.001 | 1.93 | < 0.001 | 1.97 | < 0.001 | 2.11 | < 0.001 | -3.33 | ns | -0.52 | ns |
| IL1F5 | 2.55 | < 0.001 | 1.62 | < 0.001 | 2.32 | < 0.001 | 1.79 | < 0.001 | 2.49 | < 0.001 | -3.08 | ns | -0.46 | ns |
| IL1F7 | -1.17 | 0.012 | 1.3 | 0.991 | 0.51 | ns | 1.89 | < 0.001 | 0.89 | ns | -26.82 | < 0.001 | -4.76 | < 0.001 |
| IRAK4 | -1.37 | < 0.001 | -0.44 | 0.323 | -0.93 | 0.044 | -0.34 | 0.472 | -0.89 | 0.085 | -24.91 | < 0.001 | -2.81 | < 0.001 |
| LY64 | -1.68 | < 0.001 | -2.49 | < 0.001 | -2.62 | < 0.001 | -1.46 | 0.041 | -1.87 | 0.006 | -13.89 | < 0.001 | -2.52 | < 0.001 |
| LY96 | 1.73 | 0.027 | 1.18 | 0.028 | 1.58 | 0.015 | -0.01 | ns | 0.71 | ns | 1.95 | ns | 1.15 | ns |
| MPO | -0.96 | 0.012 | -1.53 | 0.031 | -1.58 | 0.016 | -1.34 | 0.069 | -1.45 | 0.034 | -1.7 | ns | -0.55 | ns |
| TGFB2 | -0.59 | ns | -1.74 | 0.002 | -1.59 | 0.003 | -1.67 | 0.015 | -1.53 | 0.015 | -3.9 | ns | -1.02 | 0.016 |
| TLR10 | -1.5 | < 0.001 | -2.89 | < 0.001 | -2.84 | < 0.001 | -1.3 | ns | -1.65 | 0.012 | -23.41 | < 0.001 | -3.2 | < 0.001 |
| TLR6 | 1.58 | 0.002 | 1.12 | 0.018 | 1.52 | 0.004 | 1.21 | 0.021 | 1.62 | 0.005 | -1.87 | ns | -0.53 | ns |
| TLR9 | -0.64 | ns | -2.23 | 0.009 | -2.01 | 0.024 | -2.44 | 0.009 | -2.18 | 0.018 | -0.87 | ns | -0.43 | ns |
| TNFRSF18 | -1.88 | < 0.001 | -1.08 | ns | -1.61 | 0.048 | -2.25 | 0.016 | -2.54 | 0.007 | -3.26 | 0.041 | -1.37 | ns |

T(D), Tajima’s D; FL(D*), Fu and Li’s D*; FL(F*), Fu and Li’s F*; FL(D), Fu and Li’s D; FL(F), Fu and Li’s F; FW(H), Fay and Wu’s H; FW(N), normalized Fay and Wu’s test. AFR, Africans; EUR, Europeans.
